# Oxidative Stress, Bone Marrow Failure, and Genome Instability in Hematopoietic Stem Cells

**DOI:** 10.3390/ijms16022366

**Published:** 2015-01-22

**Authors:** Christine Richardson, Shan Yan, C. Greer Vestal

**Affiliations:** Department of Biological Sciences, UNC Charlotte, 9201 University City Blvd., Woodward Hall Room 386B, Charlotte, NC 28223, USA; E-Mails: shan.yan@uncc.edu (S.Y.); cvestal1@uncc.edu (C.G.V.)

**Keywords:** oxidative damage, reactive oxygen species, ROS, bone marrow failure, base excision repair, excision repair cross complement, aging, genome stability, cancer

## Abstract

Reactive oxygen species (ROS) can be generated by defective endogenous reduction of oxygen by cellular enzymes or in the mitochondrial respiratory pathway, as well as by exogenous exposure to UV or environmental damaging agents. Regulation of intracellular ROS levels is critical since increases above normal concentrations lead to oxidative stress and DNA damage. A growing body of evidence indicates that the inability to regulate high levels of ROS leading to alteration of cellular homeostasis or defective repair of ROS-induced damage lies at the root of diseases characterized by both neurodegeneration and bone marrow failure as well as cancer. That these diseases may be reflective of the dynamic ability of cells to respond to ROS through developmental stages and aging lies in the similarities between phenotypes at the cellular level. This review summarizes work linking the ability to regulate intracellular ROS to the hematopoietic stem cell phenotype, aging, and disease.

## 1. Introduction

Organisms in aerobic environments are continuously exposed to reactive oxygen species (ROS). ROS include oxygen molecules (O_2_), superoxide anion radicals (O_2_^−^), hydroxyl free radicals (OH), singlet oxygen (^1^O_2_), and hydrogen peroxide (H_2_O_2_), and can be generated by imbalanced endogenous reduction of oxygen by cellular enzymes or in mitochondrial respiratory pathway, as well as by exogenous exposure to UV or environmental damaging agents [1]. Regulation of intracellular ROS levels and ROS-mediated signaling is central maintaining the balance between self-renewal, proliferation, and differentiation of normal stem and progenitor cells in the hematopoietic and neuronal compartments, as well as the early embryonic stem (ES) cell compartment. Increases in ROS above normal concentrations can lead to oxidative stress (OS) and DNA damage [2]. OS is thought to damage approximately 10,000 bases per day per human cell and be one of the major causes of DNA damage and mutation [3]. The majority of these damaged sites are repaired through base excision repair (BER), although persistent damage or damage on two opposing strands at nearby nucleotides may result in double-strand breaks (DSBs) requiring repair by non-homologous end-joining (NHEJ) or homologous recombination (HR). OS is also known to induce higher order chromatin single strand DNA (ssDNA) nicks characteristic of those generated during programmed cell death. ROS and the DNA damage that may result are both capable of triggering multiple DNA damage response pathways. Results of signaling and cellular response to ROS range from cell proliferation and survival to growth arrest, senescence, and cell death depending on the level of ROS and cell type studied. A growing body of evidence indicates that the inability to regulate high levels of ROS leading to alteration of cellular homeostasis or defective repair of ROS-induced damage lies at the root of diseases characterized by both neurodegeneration and bone marrow failure [4,5,6,7]. Further, high levels of ROS appear to be a distinct feature of acute myeloid leukemia (AML) [8,9,10]. That these diseases may be reflective of the dynamic ability of cells to respond to ROS through developmental stages and aging lies in the similarities between phenotypes at the cellular level.

## 2. ROS Induces DNA Damage Signaling and Repair

### 2.1. Oxidative Stress and Oxidative DNA Damage

Genomes of all organisms are exposed to endogenous and exogenous stresses, such as OS. OS refers to an imbalance between antioxidant defenses and production of ROS [11,12,13,14]. ROS are generated endogenously from cellular metabolism, such as oxidative phosphorylation in mitochondria and long-chain fatty acids oxidation in peroxisomes, or exogenously by environmental toxins, such as ionizing radiation (IR), ultraviolet (UV) radiation, and DNA damaging/chemotherapeutic agents [15,16,17,18,19,20]. When ROS generation exceeds antioxidant defense capacity in cells, ROS may react with almost all macromolecules including DNA, RNA, proteins, and lipids. Notably, oxidative DNA damage may represent the major type of DNA damage, evidenced that approximately 10,000 DNA alterations are generated per mammalian cell per day [21,22,23,24]. Oxidative DNA damage includes oxidized base (purine and pyrimidine) damage, oxidized sugar moiety damage, apurinic/apyrimidinic* (*AP) sites, DNA single-strand breaks (SSBs), DSBs, DNA intrastrand crosslinks, DNA intrastrand crosslinks, DNA interstrand crosslinks (ICLs), protein-DNA crosslinks, mismatched pairs with damaged bases, stalled DNA replication forks, and oxidatively-generated clustered DNA lesions (OCDLs) [21,22,23,24,25]. OS has been implicated in the pathogeneses of multiple diseases, such as bone marrow failure, cancer, and neurodegenerative disorders [26,27,28].

### 2.2. DNA Damage Response Pathways and DNA Repair

**Figure 1:**
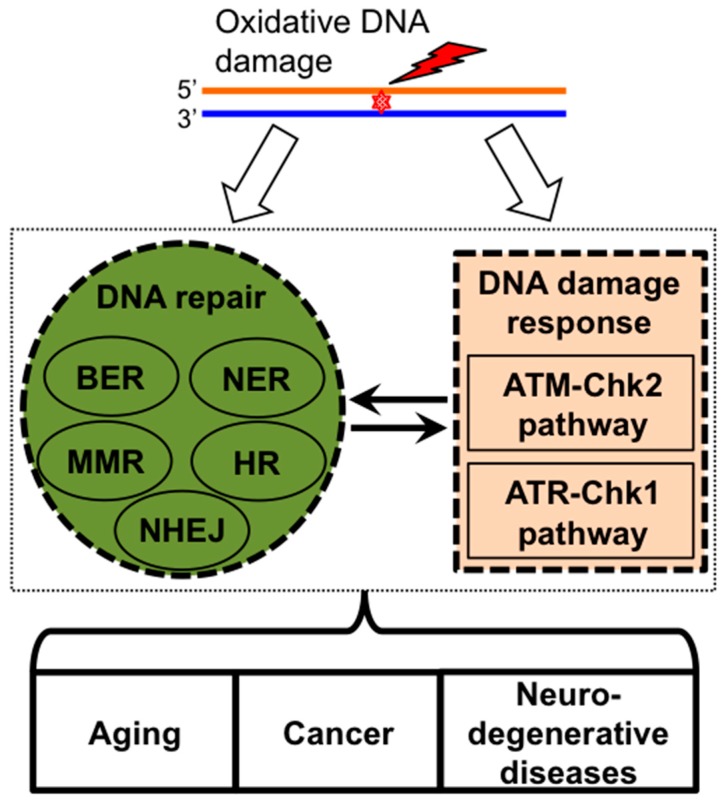
Cellular responses to oxidative DNA damage. DNA repair pathways (BER, NER, MMR, HR, and NHEJ) and DNA damage response pathways (ATM-Chk2 and ATR-Chk1) are integrating into an interacting network in response to OS. Defective DNA repair and response pathways are associated with aging, cancers, and neurodegenerative diseases. BER (base excision repair); NER (nucleotide excision repair); MMR (mismatch repair); NHEJ (non-homologous end-joining); HR (homologous recombination); ATM-Chk2 (Ataxia-telangiectasia mutated-Checkpoint kinase 2); ATR-Chk1 (ATM- and Rad3-related-Checkpoint kinase 1); OS (oxidative stress).

To maintain genomic integrity, DNA repair and DNA damage response (DDR) pathways are employed in cellular responses to oxidative DNA damage [22,23,29,30] (Figure 1). The majority of these damaged sites are repaired through BER. However, depending on the extent of oxidative DNA damage processes, such as nucleotide excision repair (NER), mismatch repair (MMR), HR, and NHEJ, may be employed in the repair processes to protect cells from OS [17,22]. Additionally, Ataxia-telangiectasia mutated (ATM)-Checkpoint kinase 2 (Chk2) and ATM- and Rad3-related (ATR)-Checkpoint kinase 1 (Chk1) checkpoints are the two major DDR pathways induced by oxidative DNA damage in order to coordinate DNA repair process with cell cycle progression, transcription, apoptosis, and senescence [23,30,31,32,33,34]. ATM modulates cell cycle in response to multiple types of cell stress and DNA damage including oxidative stress. In response to DSBs, ATM auto-phosphorylates its own Ser1981, leading to dimer dissociation into a monomer and the full activation of ATM [35,36]. ATM is also directly activated after disulfide bond-dependent conformation change induced by OS in the absence of direct DNA damage [37,38,39]. In support of a critical role for ATM in protecting hematopoietic stem cells (HSC) from OS, the HSC compartment of ATM-deficient mice present with increased levels of ROS-dependent phosphorylation of the downstream effector mitogen activated protein kinase (MAPK) p38 protein that is known to activate both cell cycle signaling and apoptosis in response to multiple cellular stressors including OS, UV, and inflammatory cytokines. This loss of ATM signaling and increase in p38 phosphorylation is in turn associated with a loss of HSC quiescence [40]. Notably, ATR is activated by oxidative DNA damage after a distinct 3'–5' SSB end resection by a BER protein APE2 [41]. Functional interplay between various DNA repair programs and ATM-Chk2- and ATR-Chk1-dependent DDR pathways in response to oxidative stress is summarized in a recent review article [28]. A chronic impairment of the DDR would be expected to result in accumulation of DNA damage, a mutator effect, and genome instability as observed in normal aging, cancer, and neurodegenerative disease (Figure 1). Elevated intracellular ROS promotes DNA DSBs and altered NHEJ repair leading to chromosomal deletions, translocations, and tumorigenesis [42,43,44]. Interestingly, multiple studies have identified elevated ROS production in the mitochondrial compartment of apoptotic cells. However, the extent of interplay, if any, between damage signaling pathways or DNA repair pathways in the mitochondrial and nuclear compartments is unclear.

## 3. Bone Marrow Failure Syndromes

Bone marrow failure is characterized by the progressive absence of one or more hematopoietic cell lineages. These syndromes are characterized as inherited or acquired. The best-characterized inherited syndromes include Fanconi anemia (FA), as well as the telomere diseases dyskeratosis conegenitia (DC), Diamond-Blackfin anemia (DBA), and Shwachman-Diamond syndrome, while the most prevalent acquired syndrome is aplastic anemia (AA). There is increasing evidence linking elevated ROS levels and impaired DNA repair mechanisms to contribute to bone marrow failure observed in these diseases. On the other hand, neurodegeneration corresponds to any pathological condition that primarily affects neurons and neurodegenerative disorders including Alzheimer disease, Parkinson disease, Huntington disease, and amyotrophic lateral sclerosis [45]. However, both systems appear particularly sensitive to the control of intracellular ROS and OS, and altered responses to these “leading to DNA damage have been implicated in the pathogeneses of both bone marrow failure and neurodegenerative disorders [4,6,7,26,27,28,46,47].

### 3.1. Fanconi Anemia

FA is an autosomal X-linked disease that is characterized mainly by bone marrow failure, anemia, developmental abnormalities, cancer susceptibility, and altered mitochondrial function. It is considered a rare disease affecting approximately 1 in every 100,000 people [48]. FA is a chronic and degenerative disorder with the probability of patients developing leukemia, squamous cell carcinomas, or liver tumors increasing by 40% by age 30, 50% by age 45 and 76% by age 60 [49]. Progressive bone marrow failure and myeloid malignancies account for 90% of deaths in FA patients. At the molecular level, there are 15 complementation groups that been identified, and mutations in any of these genes can lead to FA or a similar disorders. FA proteins that make up the core complex are FancA, FancB, FancC, FancE, FancF, FancG, FancL, and FancM, and defects in any are known to cause FA in humans. This core complex acts to ubiquitylate downstream targets FancD2 and FancI that then act in parallel with the remaining FA proteins (FancD1, FancJ, FancN, FancO, and FancP) to repair ICLs. Several mouse models have provided insight into the pathology and etiology of the disease.

There is some discordance between humans with FA and FA mouse models, most notably the absence of bone marrow failure, anemia, and developmental abnormalities in mice that are the defining characteristics of the disease in humans. These differences, however, could be in part due to human reliance on the FA pathway for detoxification and tumor suppression to ensure normal development whereas laboratory mice lack the same genotoxic stressors that may trigger the disease [50]. Almost all FA mouse models so far have exhibited reduced fertility, and cells derived from these mice display sensitivity to ICL inducing agents [50]. *FancA^−/−^* mice, for instance, do not display hematopoietic abnormalities or congenital defects; however, they have hypergonadism and mouse embryonic fibroblasts (MEFs) derived from these mice are hypersensitive to mitomycin C (MMC) [51]. Interestingly, bone marrow-derived megakaryocyte progenitors demonstrate impaired proliferation and all bone marrow cells derived from *FancA^−/−^* mice display increased apoptosis indicating a possible HSC defect [52]. Also, both *FancA^−/−^* and *FancG^−/−^* mice have increased apoptosis in neural stem and progenitor cells that leads to microcephaly [53].

*FancC^−/−^* mice born do not display overt hematopoietic abnormalities, however they are born with sub-Mendelian frequency and have a significantly increased incidence of micropthalmia in C57BL/6J mice [54]. HSC derived from *FancC^−/−^* mice have impaired differentiation and self-renewal capacity [55], a defect that is rescued by retroviral-mediated gene transfer of *FancC*, indicating that FancC is required for properly functioning HSCs. Also, *FancC^−/−^* mice exposed to low doses of MMC develop progressive bone marrow failure and chromosomal aberrations [56]. Differences in humans with FA and the mouse models may be due to the differences in environmental and dietary exposures.

*FancD1/Brca2* is downstream from the FA core complex. *Brca2* is required for HR-directed repair of DNA DSBs. *FancD1* knockout in mice leads to embryonic lethality, but a mouse model has been created with homozygous deletion of exon 27, which allows expression of the protein but prevents *FancD1* from interacting with *FancD2* [57]. *FancD1^∆27/∆27^* mice display similar phenotypes to *FancA^−/−^* and *FancC^−/−^* mice, though there is some evidence that the hematopoietic abnormalities are more severe than with the other phenotypes. For instance, *FancD1^∆27/∆27^* mice are significantly more sensitive to MMC and acquire spontaneous chromosomal aberrations and early loss of colony forming hematopoietic progenitor cells compared to the other phenotypes [58]. This severity would be expected as *FancD1* is necessary not only for ICL repair, but also for DSB repair via HR. Notably, unlike the other FA mouse models, *FancD1^∆27/∆27^* mice do not have impaired fertility indicating that it is dispensable for meiosis unlike the other FA proteins, but is important for somatic cell HR [57].

*FancD2^−/−^* mice are born with sub-Mendelian frequency and also display growth retardation, a phenotype that varies based on the genetic background of the mice. These mice have an increased incidence of tumor formation similar to that of *FancD1* haploinsufficiency [59]. Also, the phenotype of these mice is more severe than those with deleted FA core proteins, indicating alternate activity outside of the FA pathway, possibly protecting against oxidative DNA damage including DSBs [60].

Cells derived from FA mutant mice are hypersensitive to OS. It has recently been shown that impaired or reduced mitochondrial function in FA cells is coupled with increased intracellular ROS, and inactivation of enzymes essential for energy production [61]. Intriguingly these defects, as well as hypersensitivity to MMC can be partially rescued by overexpression of superoxide dismutase (SOD1) [61]. Bone marrow progenitor cells derived from *FancC^−/−^* mice undergo replicative senescence following exposure to hypoxic/hyperoxic conditions [62]. Interestingly, the *FancC^−/−^*/*Sod1^−/−^* mouse model was created to increase endogenous OS. These mice demonstrate loss of hematopoietic progenitor cells in the bone marrow leading to anemia and leucopenia [63], strongly indicating that OS is a key factor in the initiation of bone marrow failure in FA. Also, the *FancD2^−/−^*/*Aldh2^−/−^* mice, which can no longer detoxify aldehydes by oxidizing them to carboxylic acids, present with severe phenotypes including the development of acute lymphoblastic leukemia (ALL) after three to six months. Exposure to ethanol *in utero* results in development of physical abnormalities and death; and surviving mutant mice have a vast reduction in bone marrow cellularity [64]. Taken together, these models demonstrate that FA phenotypes that are hallmarks of the disease may indeed require stressors, such as aldehydes or ROS, in order to initiate the disease phenotype, and it is the absence of these stressors in the laboratory environment that prevents the FA mouse models from accurately mimicking the disease in humans.

### 3.2. Additional Degenerative Excision Repair Defects

Cockayne syndrome (CS) and xeroderma pigmentosum (XP) are characterized by defects in DNA repair of damage by crosslinking agents or elevated ROS due to mutation of any one of the excision repair pathway cross complementation (ERCC) group genes. Whereas CS patients are defective in the transcription coupled NER (TC-NER) pathway and present with neurological abnormalities, XP patients are deficient in the global genome NER (GG-NER) pathway and are hypersensitive to sun and susceptible to carcinoma [65]. Mutant mouse models have been generated for most of the proteins involved in this pathway that mimic the genotypes and phenotypes of patients with CS or XP.

CS is caused by defects primarily in *Csa* and* Csb*, which are the core proteins essential for TC-NER. *Csa^−/−^* or *Csb^m/m^* mice genetically mimic the disease, and both mutants present with UV sensitivity, loss of photoreceptors, increased sensitivity to γ-irradiation, and mild neurodegeneration [66]. Cells from *Csb^m/m^* mice but not *Csa^−/−^* mice show hypersensitivity to γ-IR and paraquat which generates superoxide anions and H_2_O_2_ in cells indicating that *Csb* plays a role in the repair of oxidative damage [67,68]. Although *Csa^−/−^* mice do not show an increased sensitivity to OS, human cells deficient in CSA are more sensitive to H_2_O_2_ than wild-type cells [69], and primary fibroblasts and keratinocytes from CSA patients are hypersensitive to potassium bromate, a known inducer of oxidative damage [70]. This indicates that the role of the CSA protein may differ between species and that it is likely involved in the oxidative damage response in humans but not mice [71].

XP is caused by defects in core NER proteins as well as proteins in the GG-NER pathway. *Xpc* functions primarily in the GG-NER pathway and *Xpc^−/−^* mice have a reduced lifespan and have a propensity for developing spontaneous tumors Also, these mice have been demonstrated to have a very large increase in lung tumor incidence compared to controls thought to be a result of chronic OS [72]. Also, fibroblasts derived from *Xpc^−/−^* mice have demonstrated OS sensitivity compared to controls by mutation accumulation and decreased survival [73]. Analysis of bone marrow cells from *Xpc^−/−^* mice shows reduced cellular viability, hypocellularity of most lineages, reduced numbers of CFU (colony-forming units), and increased sensitivity to carboplatin [74]. Since XP patients develop skin cancers by the age of 20, and the average life expectancy is early 30s, the findings in bone marrow of the *Xpc^−/−^* mice suggest the possibility that if patients were to live to later age, they could exhibit myelosuppression or bone marrow failure. In contrast to the *Xpc^−/−^* mice, *Xpa^−/−^* mice have a significant increase in tumors in the liver, but not in the lungs and do not have an increase in mutation load like the *Xpc^−/−^* mice [73]. However, cells derived from XPA patients exhibit defective repair in 8,5'-(*S*)-cyclo-2'-deoxyadenosine, a DNA lesion induced by ROS indicating that XPA may play a role in the repair of oxidative bulky lesions [75].

Cells from both CS and XP individuals affected by disease and from multiple mouse models have elevated ROS and defective mitochondrial function [65,75]. Interestingly, a similar set of defects are observed in lymphoblasts from DC patients, namely elevated ROS production, impaired DNA response leading to apoptosis and a proliferative defect [76], further connecting elevated ROS with bone marrow failure outcome.

Additional proteins in the ERCC complement group are now being identified as central to the etiology of other bone marrow failure syndromes. Through Next-Gen sequencing, ERCC6L2 was recently reported as deficient in two patient samples both presenting with bone marrow failure and neurodegeneration [77]. Loss of function of the same gene in both cases demonstrates the link between these two degenerative processes. Reduction of ERCC6L2 induced by siRNA knockdown in cells results in significantly increased intracellular ROS, effective DNA repair response shown by γ-H2AX foci, reduced cell viability and increased sensitivity to a panel of DNA damaging agents [77]. The similarities in phenotypes from ERCC genes thus far suggest that additional mutations in ERCC genes will be identified as a paradigm for bone marrow failure syndromes.

## 4. Hematopoietic Stem Cell Response to ROS

### 4.1. HSC Response to ROS

ROS are important for fate determination of normal stem cells [78]. Normal HSC are primarily in a quiescent state within the BM niche [79]. HSC exposed to elevated ROS exhibit altered characteristics and undergo both proliferation and differentiation, typically after mobilization to the oxygen rich bloodstream [80], but also to senescence and apoptosis in a dose-dependent manner [78]. This impact of ROS is evolutionarily conserved and observable in analogous *Drosophila* systems [81].

A number of transcriptional microarray, exome, and proteomic approaches have been utilized in an effort to understand the signal transduction pathways activated in response to ROS [82,83,84,85,86,87,88,89,90,91]; however, few have centered on specific HSC populations. Despite this, the p53, Akt, MAPK, and ATM pathways have all been implicated in HSC response to ROS. MDM2 promotes survival of hematopoietic progenitors by promoting p53 degradation [92]. The Forkhead box O (FoxO) family of transcription factors has known roles in metabolism, proliferation, and OS response and resistance (recently reviewed in [93]). Single FoxO3a deficiency in HSCs is associated with elevated intracellular ROS [94]. Triple knock-out *FoxO1*^−/−^ *FoxO3a*^−/−^ *FoxO4*^−/−^ mice exhibit reduced numbers of both HSC and progenitor cell populations [95]. Recent molecular analysis suggests that the co-factor KDM5 activates OS resistance genes through interaction with the lysine deacetylase HDAC4 to in turn promote FoxO deacetylation and alter specificity of target gene binding [96]. *Drosophila* also demonstrates signaling paradigms with increases in ROS signaling through JNK pathway, FoxO activation, and polycomb downregulation [81]. In conjunction with elevated ROS, depletion of the Meis1 transcription factor in mice leads to apoptosis, loss of HSC quiescence, and loss of transplantation capacity [97].

Differences in the levels of ROS also impact the observed cellular response of stem cells [98]. Elevated levels of OS induced by mutations of metabolic enzyme genes, ischemia/reperfusion, chemotherapy, or chronic exposure to 10–100 μM H_2_O_2_ induces multiple effects ranging from cell cycle arrest to death, depending on the cell type [99,100]. At submicromolar concentrations, ROS and H_2_O_2_ act as proliferation and growth signaling molecules. Low concentration exposure (100 μM) of stem cells is sufficient to promote hyperphosphorylation of Akt and RAF1, activation of insulin signaling pathways, and phosphorylation of MAPK p38 within 1 h [98]. This initial rapid growth is then temporally followed 4–24 h later by induction of cell cycle checkpoints characterized by dephosphorylation of Rb, hyperphosphorylation of cdc2, and down-regulation of E2F targets, such as *FoxD3* [98].

**Figure 2:**
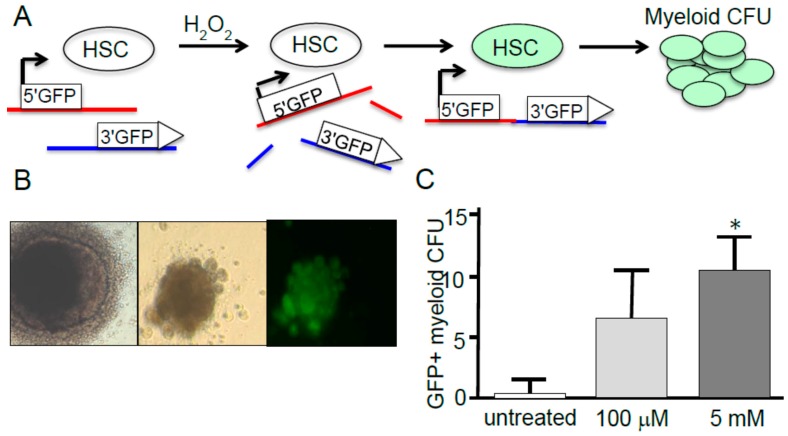
Direct connection between exposure of hematopoietic stem cells (HSC) to reactive oxygen species (ROS) and genome rearrangements. (**A**) Scheme of exposure of genetically engineered HSC to ROS and scoring of GFP+ cells indicative of translocations; (**B**) **Left** image—HSC colony by contrast microscopy (100×); **Middle** image—GFP+ derivative myeloid CFU (colony-forming units) by contrast microscopy (100×); **Right** image—Same GFP+ CFU colony shown in middle by fluorescence microscopy (100×); and (**C**) Bar graph showing dose dependent appearance of GFP+ myeloid CFU derived from HSC exposed to 0, 100 μM, or 5 mM H_2_O_2_ for 30 min, then returned to normal conditions. 100 μM resulted in an average frequency of CFU at 6.5 × 10^−6^; 5 mM resulted in an average frequency of CFU at 10.4 × 10^−6^. * denotes statistically significant stimulation of chromosomal translocations by H_2_O_2_ (students *t*-test *p*-value < 0.037). A few colonies were observed in untreated samples that by contrast microscopy but appeared to be unviable and auto-fluorescent rather than *bona fide* GFP+ CFU; however, further analysis was not performed on them so they are reported here.

To directly link ROS to genetic instability in HSC, we genetically engineered murine HSC cells to contain two transgene reporters at unlinked loci—each with either a 5' or 3' exon of the green fluorescent protein (GFP). This scheme enables us to score for chromosomal translocations following DNA damage and repair by interchromosomal NHEJ repair that results in a repair event joining the two exons onto the same DNA helix to form an intact GFP (Figure 2A). Following exposure of the engineered HSC to H_2_O_2_ (Figure 2A, Figure 2B left image), surviving cells were then allowed to proliferate into myeloid CFU by standard protocols [101] (Figure 2B middle image), and those CFU expressing GFP readily identified by fluorescent microscopy (Figure 2B right image). This approach demonstrated that a single 30 min exposure of HSC to 100 μM H_2_O_2_ or 5 mM H_2_O_2_ is sufficient to promote the appearance of GFP+ colonies in a dose-dependent manner at average frequencies of 6 × 10^−6^ and 10 × 10^−6^, respectively (Figure 2C). This* in vitro* system is analogous to the outgrowth of transformed myeloid leukemic cells and connects ROS and genome rearrangements in HSC and leukemia.

It is now well established that IR promotes differentiation, short-term apoptosis, and long-term senescence of hematopoietic stem cells [78,102]. Several studies demonstrated that IR of CD34+ hematopoietic progenitor cells induces indirect effects on DNA damage through elevated OS [102,103]. IR of CD34+ cells induces early increase of superoxide anion radicals and hydrogen peroxide, followed by elevated MnSOD expression by 24 h, followed by elevated persistent intracellular ROS over longer culture periods associated with reduced clonogenic potential [104,105]. By contrast γ-H2AX expression and foci associated with direct DNA DSBs are elevated in short periods following IR but return to baseline by 24 h [105]. Presence of insulin-like growth factor 1 (IGF-1) in irradiated CD34+ cells can inhibit the mitochondria-mediated apoptosis and rescue the clonogenic potential defect [104]. This role for IGF-1 in response to ROS mirrors transcriptional and proteomic profiling of stem cells demonstrating induction of insulin regulated pathways in response to ROS [98]. Further, sublethal total body IR of mice leads to a persistent elevation of ROS in HSC associated with sustained increases in oxidative DNA damage and DSBs, inhibition of HSC clonogenic potential, and induction of HSC senescence, at least in part due to upregulation of nicotinamide adenine dinucleotide phosphate oxidase 4 (NOX4) [103].

### 4.2. HSC Response to ROS and Aging

Control of ROS in ES cells is now understood to be critical to maintenance of self-renewal phenotype. Molecular evidence suggests this is due, at least in part, to ROS activation of miR-29b that targets Sirt1 to control ES cell self-renewal [106]. Differentiation of ES cells leads to increased intracellular ROS [61,107]. Interestingly, during differentiation of ES cells, superoxide production, cellular levels of intracellular ROS, and DNA damage levels increase although expression of major antioxidant genes and genes involved in multiple DNA repair pathways are downregulated [107], and DNA repair by HR is reduced [101].

Aging is associated with reduced expression of multiple DNA repair proteins, reduced DNA repair efficiency and elevated levels of genome instability in hematopoietic cells [6,108,109,110]. Hematopoietic cells in older mice and humans are characterized by reduced regenerative potential, lineage bias toward myeloid cells, and increased potential for outgrowth of genetically unstable cells and malignancy. Serial transplantation of human HSC into immunodeficient mice is associated with replication stress, elevated intracellular ROS, accumulation of DNA damage, and reduced self-renewal potential that can be further aggravated by addition of a glutathione synthetase inhibitor [80]. The connection between natural HSC aging and intracellular ROS was made by the ability to separate the CD34−, Lin− (lineage cocktail negative), Sca-1+, c-kit+ HSC compartment into two fractions based on intracellular ROS levels [111], and the observation that the ROS^low^ fraction has higher self-renewal capacity and serial transplantation capability. Conversely, ROS^hi^ fraction has reduced capacity for self-renewal and in addition is biased toward myeloid differentiation, similar to aged mice* in vivo* [111]. Intriguingly, the observed phenotype can be partially rescued following inhibition of MAPK p38 suggesting that p38 is a potential therapeutic target [40,111].

ROS seem well tolerated in “young” cells with highly proficient DNA repair, and thus have few if any long-term deleterious effects. However, “older” cells with less efficient repair become more sensitive to ROS levels, which lead to increased genome instability. Recent work demonstrated that quiescent HSC (analogous to the ROS^low^ fraction) repair DNA damage less efficiently than progenitors leading to accumulation of DNA damage and impaired function, suggesting a mechanism for increased presentation of myeloid malignancies in aged populations [112].

HSC reactivity to ROS during aging* in vivo* combines cell-intrinsic and cell-extrinsic mechanisms [113]. A relationship between the HSC compartment and supporting cells within the bone marrow niche is central to homeostasis and changes during not only aging but also other pathophysiologic processes including atherosclerosis, hypertension, and diabetes [114,115]. HSC and progenitors are typically exposed to low levels of ROS in the bone marrow niche, and hypoxia in turn helps preserve HSC and progenitor characteristics. HIF1α, HIF2α have been shown to be major regulators in modulating the response of HSC to ROS in this microenvironment [103,116,117,118]. ROS derived from NAPH oxidase and mitochondria in response to injury and stress is elevated in supportive bone marrow microenvironment cells, leading to hypoxia and HIF1α expression, and HSC progenitor expansion and differentiation [114]. Similarly, HIF2α depletion in HSC leads to increased ROS, activation of endoplasmic reticulum (ER) stress and apoptosis [119].

### 4.3. Leukemic Cells and ROS

Defective signaling in response to ROS occurs in multiple leukemic stem cell populations to promote proliferation, differentiation, genomic, and epigenetic alterations, immune evasion, and survival [120]. ROS act as signaling molecules to regulate redox-sensitive transcriptional factors, enzymes, oncogenes, and other downstream effectors, and thus AML cells are thought to be addicted to elevated ROS [120]. Extracellular ROS, but not intracellular mitochondrial ROS, have been found to be significantly elevated in both AML cell lines and cells derived from patients [8,9,10] and correlates with constitutive activation of NOX, reduced levels of glutathione, and attenuated p38 MAPK responsiveness, all postulated to promote proliferation of AML blasts [121]. AKT negatively regulates the FoxO proteins, which target expression of several anti-oxidant enzymes, including the GPx, catalase (CAT) and SODs. Akt is constituitively activated in most primary AML cells, leading to FoxO inhibition, and increased ROS levels [95,122]. Activated RAS is a feature of a portion of AML patients, and upregulates ROS levels through NOX to promote proliferation of hematopoietic cells [121]. Similarly, FLT3 plays a role in H_2_O_2_ production in AML cells through NOX p22 protein, while knockdown of p22 leads to immediate reduction of H_2_O_2_ within 24 h [123]. Activating mutations of FLT3 by internal tandem duplications or activating mutations of the FLT3 receptor are seen in a large percentage of AML patients associated with increased DNA DSBs, genome instability, and poor prognosis [124]. Interestingly FLT3 activation in AML cells leads to inactivation of tumor suppressor PTP, DEP1, and reactivation of DEP1 is sufficient to decrease ROS. Elevated ROS DNA damage occurs both in the nuclear fraction and mitochondria. Mitochondria have more limited DNA repair functions and mitochondrial DNA genome instability has been noted in AML [125]. HIF2α activity may also promote proliferation and inhibition of apoptosis of AML blasts in response to ROS [119].

Philadelphia-negative chronic myeloproliferative neoplasms (MPNs; essential thrombocythemia, polycythemia vera, and myelofibrosis) have recently been shown to be associated with chronic inflammation, OS and accumulation of ROS. Transcriptional profiling of these revealed several OS modulating genes significantly deregulated in MPNs [126]. Interestingly the transcriptional factor Nrf2, which is upregulated in multiple AML cells, was found to be downregulated in this study [126,127]. In addition, downregulation of ATM and p53 were also observed, although the impact of these is likely to be broader than through OS signaling. OS may also play a role in mutation accumulation and progression of myelodysplastic syndromes (MDS) to AML as suggested by studies in a mouse model of MDS that displays increased ROS in Lin− bone marrow cells, increased DNA breaks, and increased mutation frequency over time, although to date these studies have been correlative and not causative [128].

Elevated ROS in in AML cells may be exploited as a therapeutic strategy. A recent report outlines the design of an H_2_O_2_ activatable anti-AML agent based on a conjugate addition strategy [129]. In this, a dual pharmacophore was developed in which the first is a peroxidase acceptor and the second is a pendant amine which react via conjugate addition following H_2_O_2_ oxidation within the tumor cell, and demonstrated 10-fold increased activity of the pendant amine agent in AML cells over normal CD34+ cells.

## 5. Conclusions

A growing body of evidence indicates that the inability to regulate high levels of ROS leading to alteration of cellular homeostasis or defective repair of ROS-induced damage lies at the root of diseases characterized by both neurodegeneration and bone marrow failure [4,5,6,7,26,27,28,46,47]. That these diseases may be reflective of the dynamic ability of cells to respond to ROS through developmental stages and aging lies in the similarities between phenotypes at the cellular level. It appears that neuronal or hematopoietic progenitors are particularly susceptible to altered ROS and mutation of any gene within these systems that alters ROS levels is more likely to trigger signaling pathways to promote cell death and hypocellularity within the tissue compartments. Natural aging in the human population is associated with multiple neurodegenerative phenotypes as well as a loss of the HSC population, genome instability, and cancer. These outcomes are likely also reflective of the sensitivity of progenitors in both these compartments to ROS homeostasis. The coupled reduction of DNA repair protein expression and DNA repair efficiency in neuronal stem and HSC compartments would also put these cells at higher risk. However, why these two organ systems appear to be particularly susceptible to ROS remains unclear and warrants further study. It is intriguing that several recent studies have demonstrated some amelioration to elevated ROS levels through add-back of DNA repair proteins to diseased cells; whether these will be harnessed for personalized medicine remains to be seen.
